# NOP56 promotes hepatocellular carcinoma progression through 2′-O-methylation

**DOI:** 10.1016/j.gendis.2024.101387

**Published:** 2024-08-08

**Authors:** Shuxin Yang, Juan Liu, Shengxin Luo, Wentao Wang, Jingxiang Xu

**Affiliations:** a School of Basic Medicine, Gannan Medical University, Ganzhou, Jiangxi 341000, China; b Centre for Molecular Pathology, Gannan Medical University, Ganzhou, Jiangxi 341000, China

Hepatocellular carcinoma (HCC) is a common malignant tumor and one of the leading causes of cancer-related deaths, with the fourth-highest incidence rate and the second-highest mortality rate in China.^1^ Currently, HCC is treated by surgery, radiotherapy, chemotherapy, targeted therapy, and immunotherapy. Despite advances in treatment, the prognosis of HCC remains poor, and research on the discovery of novel drug targets is continuously needed.^2^

Nucleolar protein 56 (NOP56) is an essential nucleolar protein, along with fibrillarin (FBL), nucleolar protein 58 (NOP58), small nuclear ribonucleoprotein 13 (SNU13), and small nucleolar RNAs (snoRNAs) forms the box C/D small ribonucleoprotein complexes (Box C/D snoRNPs), and these complexes guide and catalyze the 2′-O-methylation of specific nucleotides. 2′-O-methylation is a post-transcriptional modification where a methyl group (–CH3) is added to 2′ hydroxyl (−OH) of the ribose moiety, and it can occur on any base of RNA.^3^ 2′-O-methylation of rRNA can alter the assembly and function of ribosomes and affect cancer occurrence and development by regulating protein synthesis.^4^ Recent studies have revealed that 2′-O-methylation as a post-transcriptional regulation mechanism affects mRNA stability and protein expression of cancer pathways.^5^ Our previous study has indicated that NOP56 expression is significantly higher in tumor tissue than in normal tissue of HCC patients. However, the biological functions and molecular mechanisms of NOP56 in HCC remain unclear. This study demonstrates that NOP56 promotes HCC cell proliferation by regulating 2′-O-methylation and may be a potential drug target for HCC.

To validate the differential expression of NOP56 between tumor tissue and adjacent non-tumor tissues, The Cancer Genome Atlas (TCGA) database and GSE14520 dataset were used to analyze the differential expression of NOP56 in HCC, and the results showed that the mRNA expression of NOP56 was significantly higher in tumor tissue (Fig. 1A; Fig. S1A). Kaplan–Meier survival analysis showed that high expression of NOP56 was associated with shorter overall survival and disease-free survival in HCC patients (Fig. 1B; Fig. S1B). In addition, NOP56 expression in stage III-IV HCC patients was significantly higher than that in stage I-II HCC patients, and the differential expression was also observed between low grades and high grades in HCC (Fig. 1C; Fig. S1C). Furthermore, TCGA data of HCC was divided into high and low NOP56 expression groups for differential analysis, and 1484 up-regulated genes and 853 down-regulated genes (*P* < 0.05 and |log fold change| ≥ 1) were identified (Fig. S2A). Gene set enrichment analysis was used to identify pathways associated with NOP56, and the results showed that NOP56-related differential expression genes were mainly enriched in ribonucleoprotein complex biogenesis and rRNA processing pathways (Fig. 1D; Fig. S2B).

**Figure 1:**
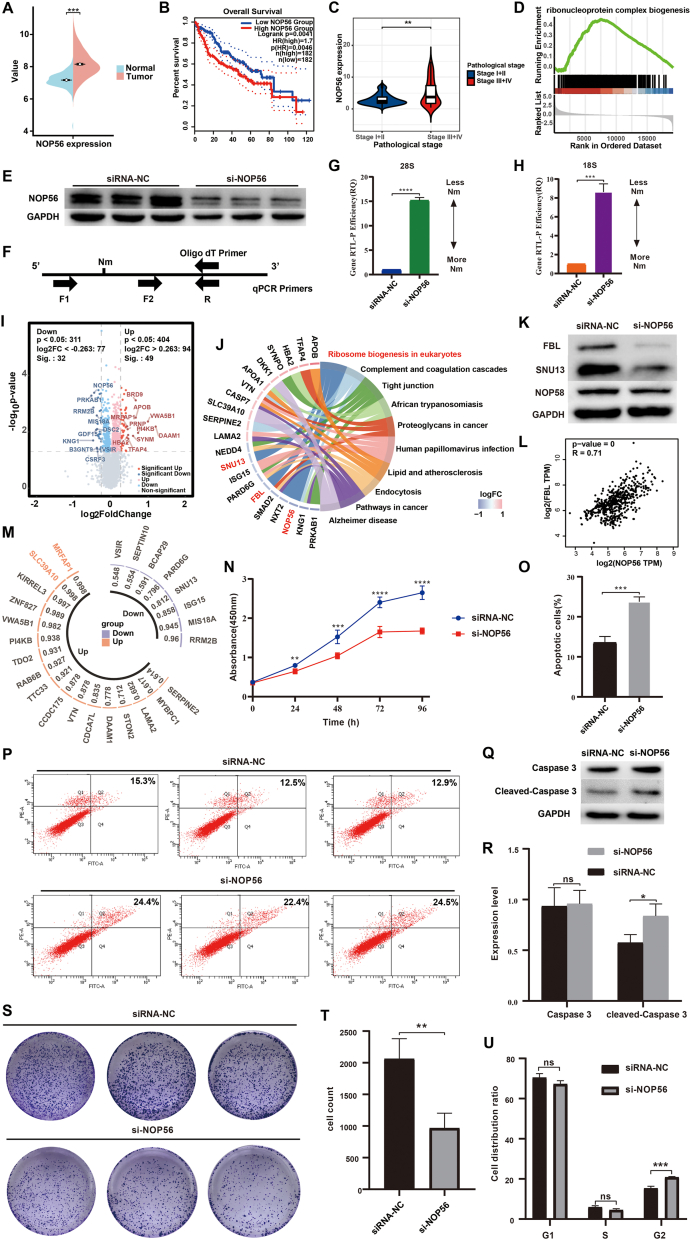
NOP56 regulates cell proliferation and apoptosis through 2′-O-methylation in hepatocellular carcinoma. **(A)** Differential expression of NOP56 in tumor tissue and normal tissue of hepatocellular carcinoma patients. The data was derived from the GSE14520 datasets. **(B)** Survival analysis of NOP56 in hepatocellular carcinoma using the GEPIA 2 website. **(C)** The differential expression of NOP56 in different pathological stages. The data was from The Cancer Genome Atlas (TCGA) dataset. **(D)** Gene set enrichment analysis of differentially expressed gene sets between NOP56-High and NOP56-Low groups. **(E)** The knockdown efficiency of NOP56 was detected by Western blot. **(F)** Schematic diagram of primer design for RTL-P assay. **(G, H)** Levels of 2′-O-methylation of 28S rRNA and 18S rRNA after NOP56 knockdown using RTL-P assay. **(I)** DEP volcano plot between the si-NOP56 group and siRNA-NC group. **(J)** Kyoto Encyclopedia of Genes and Genomes (KEGG) enrichment analysis of the DEPs. **(K)** FBL, NOP58, and SNU13 expression were detected by Western blot after NOP56 knockdown. **(L)** Correlation analysis of NOP56 with FBL expression by GEPIA 2 website. **(M)** 2′-O-methylation sites of DEPS were predicted through the DeepOMe website. **(N)** Cell proliferation of HepG2 cells between the si-NOP56 group and siRNA-NC group. **(O, P)** Analysis of cell apoptosis rate by flow cytometry between the si-NOP56 group and siRNA-NC group. **(Q, R)** Protein expression of caspase 3 and cleaved-caspase 3 between the si-NOP56 group and siRNA-NC group. **(S, T)** Cell colony forming ability between the si-NOP56 group and siRNA-NC group. **(U)** The cell cycle distribution between the si-NOP56 group and siRNA-NC group. DEP, differentially expressed protein; DFS, disease-free survival; FBL, fibrillarin; NOP56, nucleolar protein 56; NOP58, nucleolar protein 58; OS, overall survival; RTL-P, reverse transcription at low deoxy-ribonucleoside triphosphate concentrations followed by PCR; SNU13, small nuclear ribonucleoprotein 13.

Box C/D snoRNPs are mainly involved in 2′-O-methylation modifications. To investigate whether NOP56 as a core protein of box C/D snoRNPs regulated the 2′-O-methylation modifications of rRNA in HCC, specific siRNAs were designed to knock down the expression of NOP56 in HepG2 cells. Western blotting and quantitative PCR confirmed that protein expression levels of NOP56 were remarkably lower in the si-NOP56 group compared with the siRNA-NC group (Fig. 1E; Fig. S3A). The levels of 2′-O-methylation at Am2388, Um2402, Cm2409, and Gm2411 in human 28S rRNA and Am1490 in human 18S rRNA were analyzed by reverse transcription at low deoxy-ribonucleoside triphosphate concentrations followed by PCR (RTL-P) between si-NOP56 and siRNA-NC groups and the results showed a significant reduction in 2′-O-methylation level (*P* < 0.05) after NOP56 knockdown (Fig. 1F–H).

Proteomic analyses were also performed between si-NOP56 and siRNA-NC siRNA-treated HepG2 cells. A total of 81 significantly differentially expressed proteins were identified, in which 49 were up-regulated and 32 were down-regulated (|LogFC| > 0.263, *P* < 0.05), and top 10 differentially expression proteins were shown in the volcano plot (*P* < 0.05) (Fig. 1I). Kyoto Encyclopedia of Genes and Genomes (KEGG) pathway and Gene Ontology (GO) term enrichment analysis revealed a significant enrichment in the eukaryotic ribosome biogenesis pathway and the box C/D RNP complex pathway (Fig. 1J; Fig. S3B), and both the pathways include NOP56, FBL, and SNU13 proteins, indicating that knocking down NOP56 significantly affects other core proteins in box C/D snoRNPs. Western blot confirmed the decrease of FBL, NOP58, and SNU13 in the si-NOP56 group, which indicated that NOP56 knockdown influenced the stability of box C/D RNP complex (Fig. 1K; Fig. S3C). Furthermore, gene correlation analysis using the GEPIA 2 showed that NOP56 was positively correlated with FBL, NOP58, and SNU13 (Fig. 1L; Fig. S3D, E). Then, the DeepOMe website was used to predict the 2′-O-methylation modification sites of the 81 differentially expressed proteins, and 25 of these differentially expressed proteins have predicted sites of 2′-O-methylation (Fig. 1M). Among them, solute carrier family 39 member 10 (SLC39A10) and mortality factor 4 family associated protein 1 (MRFAP1) showed the highest predicted site scores, and their predicted sites are shown in Figure S4A, B.

To further investigate the biological function of NOP56 in HCC, we performed cell proliferation, apoptosis, and wound healing assay. The CCK8 assay showed that cell proliferation was significantly decreased by the knockdown of NOP56 in HepG2 cells (Fig. 1N). Apoptosis assay showed that the percentage of apoptotic cells in the si-NOP56 group was significantly higher than that in the siRNA-NC group (23.8% versus 13.6%) (Fig. 1O, P). Caspase 3 is responsible for most proteolysis during apoptosis, and the expression level of caspase 3 and cleaved-caspase 3 in the si-NOP56 and siRNA-NC groups were analyzed by Western blot. Of note, while there was no difference in the expression of caspase 3 between these two groups, the expression of cleaved-caspase 3 was significantly higher in the si-NOP56 group (*P* < 0.05) (Fig. 1Q, R). Colony formation assay showed that NOP56 knockdown effectively inhibited colony formation (Fig. 1S, T) and further demonstrated that NOP56 expression is closely related to HCC cell proliferation. NOP56 knockdown also significantly increased the proportion of HepG2 cells at the G2 phase compared with the control group, indicating that NOP56 knockdown induces cell cycle arrest at the G2 phase (Fig. 1U; Fig. S5A). However, there are no significant effects on the migratory ability of HepG2 cells after NOP56 knockdown (Fig. S5B, C).

In summary, our study demonstrates that NOP56 as a core protein of box C/D snoRNPs is a poor prognostic marker for HCC, it has an important role in regulating the formation and function of the box C/D snoRNPs complex, and its knockdown reduces 2′-O-methylation modification of rRNA and inhibits cell proliferation and promotes cell apoptosis of HCC. These results provide insights into the important role of NOP56 in regulating HCC's development and contribute to a deeper understanding of 2′-O-methylation modification molecular mechanisms in HCC, which may be potential drug targets for HCC.

## Funding

The project was funded by the High-level Talent Research Funds of Gannan Medical University (No. QD202122).

## CRediT authorship contribution statement

**Shuxin Yang:** Conceptualization, Formal analysis, Methodology, Writing – original draft, Writing – review & editing. **Juan Liu:** Methodology, Writing – original draft. **Shengxin Luo:** Methodology, Software. **Wentao Wang:** Software. **Jingxiang Xu:** Conceptualization, Data curation, Formal analysis, Funding acquisition, Investigation, Project administration, Supervision, Writing – review & editing.

## Conflict of interests

The authors declared no competing interests.
