# Supplementary material for: NOP56 promotes hepatocellular carcinoma progression through 2′-O-methylation
Source: Genes Dis. 2024 Aug 8;12(2):101387. doi: 10.1016/j.gendis.2024.101387 (PMC11605344; doi:10.1016/j.gendis.2024.101387)
Supplement: Multimedia component 1 [file mmc1.doc]

**Materials and Methods**

**Data Collection and Processing**

Liver hepatocellular carcinoma (LIHC) RNA-sequencing data and its corresponding clinical information were downloaded from TCGA database (https://gdc.cancer.gov/).The primary HCC tumor tissues and adjacent non-tumor tissues of datasets GSE14520 was obtained from the GEO database (https://www.ncbi.nlm.nih.gov/geo/), including 220 adjacent tumor tissues and 225 tumor tissues. TCGA data were divided into high and low groups based on the expression level of NOP56, and then used for analysis of differences expression and GO and KEGG enrichment. Additionally, survival prognostic analyses and gene correlation analyses were performed through the GEPIA2 website ([http://gepia2.cancer-pku.cn/#index](http://gepia2.cancer-pku.cn/#index)). All datasets were derived from unidentified patient databases.

**Cell Culture and RNA interference**

HepG2 cells (Shanghai Institute of Cell Biology, Shanghai, China) were cultured in DMEM medium supplemented with 10% fetal bovine serum , and 1% penicillin and streptomycin at 37°C in a 5% CO2 atmosphere. NOP56 siRNA and control siRNA (Ribobio, Guangzhou, China) were used in NOP56 knockdown assay. The mRNA sequence targeting NOP56 is GAGTGGGTACGGGTATCACT. The cells were transfected with GP-transfect-Mate transfection reagent (Gene Pharma, Shanghai, China) according to the procedure of manufacturer. Cells were collected after 24h or 48h of transfection, and the knockdown efficiency was verified by qPCR and Western blot.

**qPCR and rRNA methylation quantification by RTL-P**

The total RNA isolation was prepared using Trizol (Invitrogen, CA, USA). The SuperScript™ III First-Strand Synthesis System (Thermo Fisher, MA, USA) was employed for reverse transcription and cDNA synthesis. The forward primer for NOP56 was 5’-TCTCTATGCGTGTCAGGGAGT-3’, and the reverse primer for NOP56 was 5’-TGGGCAAGACGGCAGTATGT-3’. The relative expression levels were calculated using the 2^-ΔΔCt method. RT was performed in a 20 μL reaction mixture containing 2ug of total RNA, specific RT primers (10 uM) and a low (10μM) or high (10mM) concentration of dNTP. For 28S rRNA, forward primers were 28S-Fu2: 5’-AGAACTTTGAAGGCCGAAGTGG-3’ and 28S-FD1: 5’-TTGAACATGGGTCAGTCGGTCC-3’, with a reverse primer 28S-R: 5’-ATCGGTCGCGTTACCGCACT-3’.For 18S rRNA, forward primers were 18S-FU: 5’-CACCCGAGATTGAGCAATAACA-3’ and 18S-FD1: 5’-TACACTGACTGGCTCAGCGTG-3’, with a reverse primer 18S-R: 5’-GCTTATGACCCGCACTTACTGG-3’.^1^ We used RTL-P to measure site-specific rRNA methylation as described previously with minor modification.^2^ Prediction of 2'-O-methylation sites at the mRNA level of downstream genes was performed by the 2'-O-methylation site prediction web server DeepOMe (http://deepome.renlab.org/#/home) based on a hybrid CNN and BLSTM architecture.^3^

**Western blot and Proteomic analysis**

Cells were harvested and lysed by RIPA buffer (supplemented by phosphatase and protease inhibitors) and centrifuged at 10,000× g for 5 min（Solarbio, Beijing, China). Protein samples were separated electrophoretically by SDS-PAGE, and transferred to PVDF membranes (Millipore, MA, USA). The membranes were blocked for 1h in Tris-buffered saline-Tween 20 (TBST) with 5% non-fat milk. Then, immunoblottings were performed with primary antibodies (1:1000 dilution) overnight at 4°C. After washing for three times, the membranes were further incubated with secondary antibodies (1:5000 dilution) at room temperature for 1h. The signals were developed using ECL chemiluminescence reagent kit (Proteintech, IL, USA), captured by a Bio-Rad imaging system and ImageJ software was employed for image analysis. For proteomics analysis, peptides were introduced into the samples at a flow rate of 300 nL/min and uploaded onto an analytical column Acclaim PepMap RSLC, 75 μm×50 cm (RP-C18, Thermo Fisher) for separation. Mobile phase A phase: ACN-H2O-FA (99.9:0.1, v/v); mobile phase B phase: ACN-H2O-FA (80:19.9:0.1, v/v/v). Gradient elution conditions: 0~40 min, 2-28% B; 40~60 min, 28-42% B; 60~65 min, 42-90% B; 65~75 min, 90% B. The primary MS mass resolution was set to 60000, the automatic gain control value was set to 1e6, and the maximum injection time was 50 ms; the mass spectral scan was set to the full-scan charge-to-mass ratio m/z range of 350-1500, and all MS/MS pattern acquisitions were accomplished using high-energy collisional cleavage in the data-dependent positive-ion mode, with the collisional energy set to 36. The MS/MS resolution was set to 15000, with the automatic gain control set to 1e5, and the maximum injection time of the ions was 48 ms (Luming biological technology,Shanghai,China).

**Cell Proliferation, Apoptosis and Wound Healing Assay**

The Cell Counting Kit 8 (CCK-8) assay (GLPBIO, CA, USA) and colony formation assay were used for assessing the cell proliferation ability. Equal numbers of non-transfected cells and transfected cells were plated into 96-well plates with 5 replicate wells for each group. At the same time of next day, 10% CCK-8 reagent (GLPBIO, CA, USA) was added to each well and the cells were incubated for 2 h. The absorbance at 450nm was measured. For the colony formation assay, transfected HepG2 cells were plated into six-well plates containing DMEM complete medium. Cells were cultured for 8–10 days at 37 °C in a humidifed atmosphere with 5% CO2. Freshmedium was replaced every 3–4 days. After colonies have grown, treat with 4% formaldehyde for 15 minutes. The plates were then washed twice with cold PBS. Staining was performed with 0.1% crystal violet for 15 min at room temperature and analysed by ImageJ.

After 24 hours of transfection, the cells were harvested using trypsin without phenol red, centrifuged at 1000g for 5 minutes, the supernatant was discarded, and the cell pellet was washed once with 1 ml cold PBS. The cells were fixed in 1 ml cold ethanol and stored at 4°C for at least 2 hours or overnight. Then stained with propidium iodide (PI) staining solution, incubated at 37°C in the dark for 30 minutes. For the apoptosis assay, cells transfected for 48 hours were collected without EDTA-containing trypsin and washed with 1ml cold PBS. The cells were then resuspended in 1× binding buffer, adjusted to a concentration of 1-5 × 10^6/ml, and 100 µl of cell suspension was mixed with 5 µl Annexin V/FITC. After a 5-minute incubation in the dark at room temperature, 5 µl propidium iodide (PI) solution and 400 µl PBS were added. FACSCanto II flow cytometer was used to determine the cell cycle and apoptotic rate of the stained cells

For the wound Healing assay, negative control and transfected cells were maintained in the 6-well plates to form confluent cell monolayer. When the cells grew to 100% confluence, a 200 µL sterile gun head was utilized to scratch across the surface of the well. The medium was then replaced with serum-free culture medium, photographs were taken at 24 and 48 hours.

**Statistical Methods**

Western blot bands and flow cytometry results for apoptosis were analyzed using Image J and Flowjo, respectively. Statistical data analysis was performed using GraphPad Prism 8. Each experiment was conducted at least three times, and data were expressed as mean ± standard deviation (SD). Statistical analysis was performed using unpaired t-tests or analysis of variance (ANOVA). A significance level of P < 0.05 was considered statistically significant.

**References**

1. Z. W. Dong, P. Shao, L. T. Diao, et al. RTL-P: a sensitive approach for detecting sites of 2'-O-methylation in RNA molecules. Nucleic Acids Res. 2012;40(20): e157.

2. B. A. Elliott and C. L. Holley. Assessing 2'-O-Methylation of mRNA Using Quantitative PCR. Methods Mol Biol. 2021;2298:171-184.

3. H. Li, L. Chen, Z. Huang, et al. DeepOMe: A Web Server for the Prediction of 2'-O-Me Sites Based on the Hybrid CNN and BLSTM Architecture. Front Cell Dev Biol. 2021;9:686894.


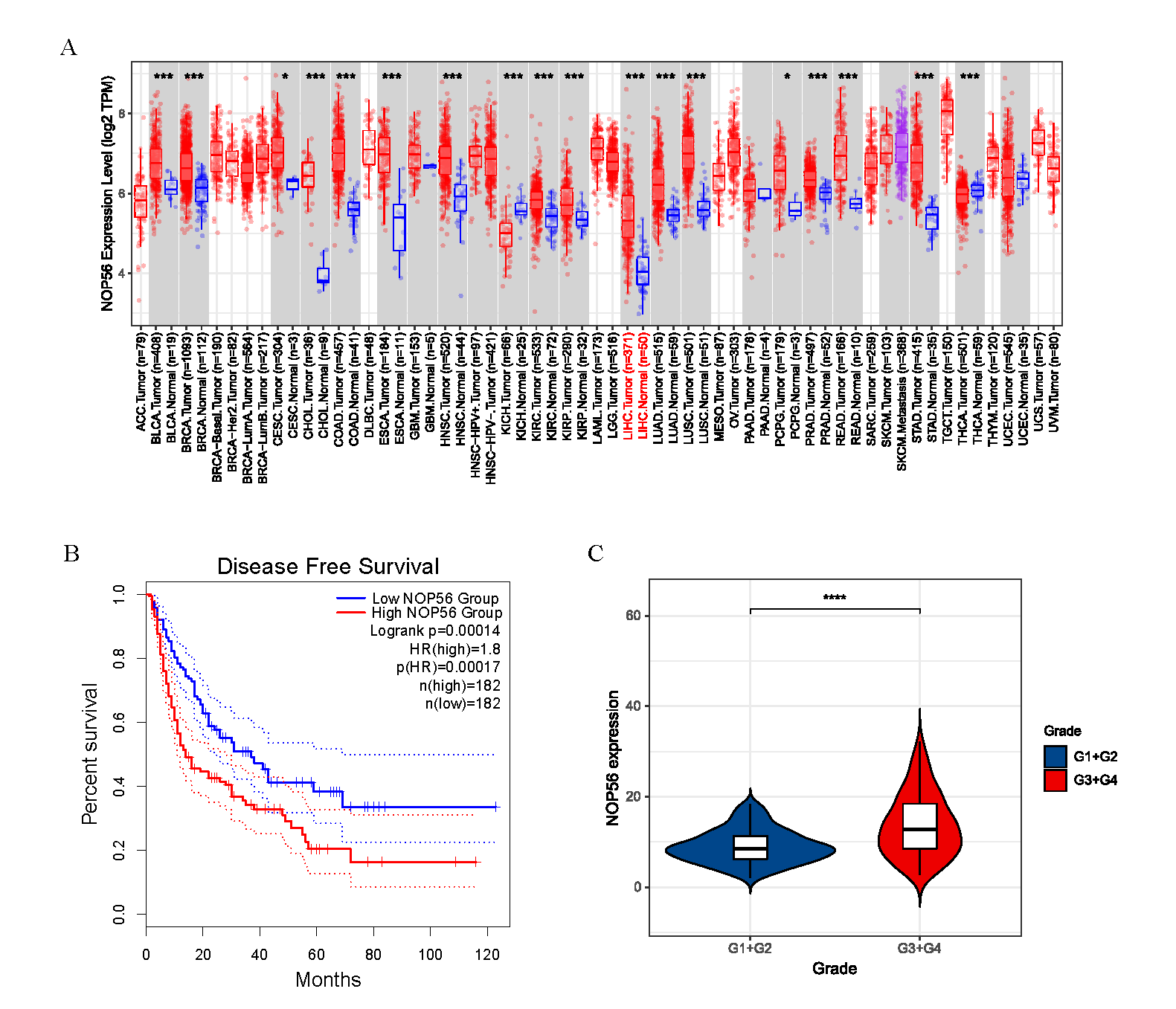


**Figure S1. High expression level and poor prognosis of NOP56 in HCC. (A)** The expression of NOP56 in multiple human cancers was visualized using the TIMER database. **(B)** Survival analysis of NOP56 in HCC using the GEPIA 2 website. DFS: diseases free survival. **(C)** The expression levels of NOP56 in tumor grade was obtained from the TCGA dataset.


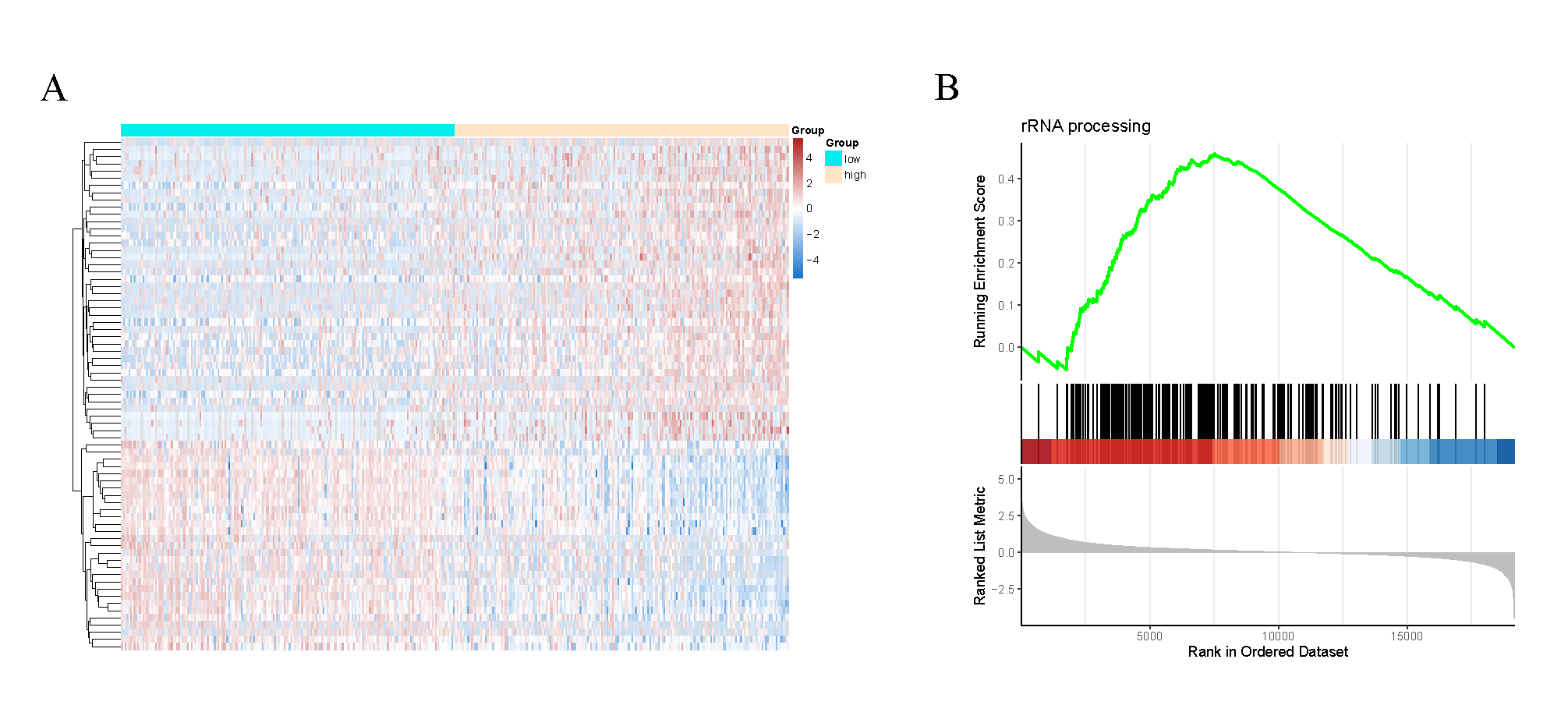


**Figure S2. Differentially Expressed Genes (DEGs) and Enrichment Analyses. (A)** DEGs heatmap between the high and low NOP56 expression groups from the TCGA-LIHC datasets. **(B)** GSEA pathway enrichment analysis of differentially expressed gene sets using TCGA-LIHC data.

**
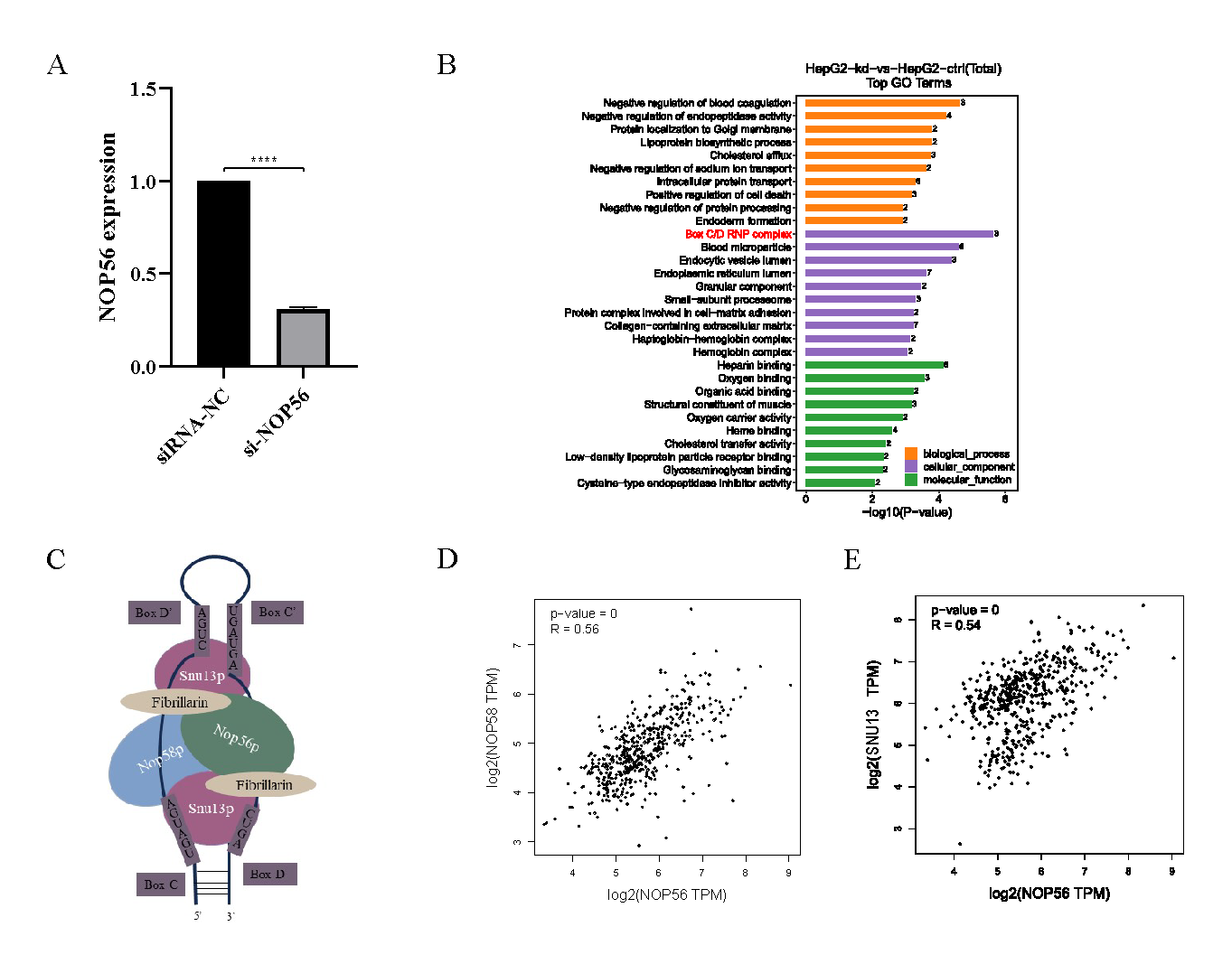
**

**Figure S3. Influence of NOP56 on box C/D snoRNPs. (A)** Histogram of knockdown efficiency of NOP56 detected by qPCR. **(B)** GO enrichment analysis of the DEPs. **(C)** Schematic diagram of the structure of box C/D snoRNPs. **(D, E)** Correlation analysis of NOP56 with NOP58 and SNU13 expression by GEPIA 2 website.

**
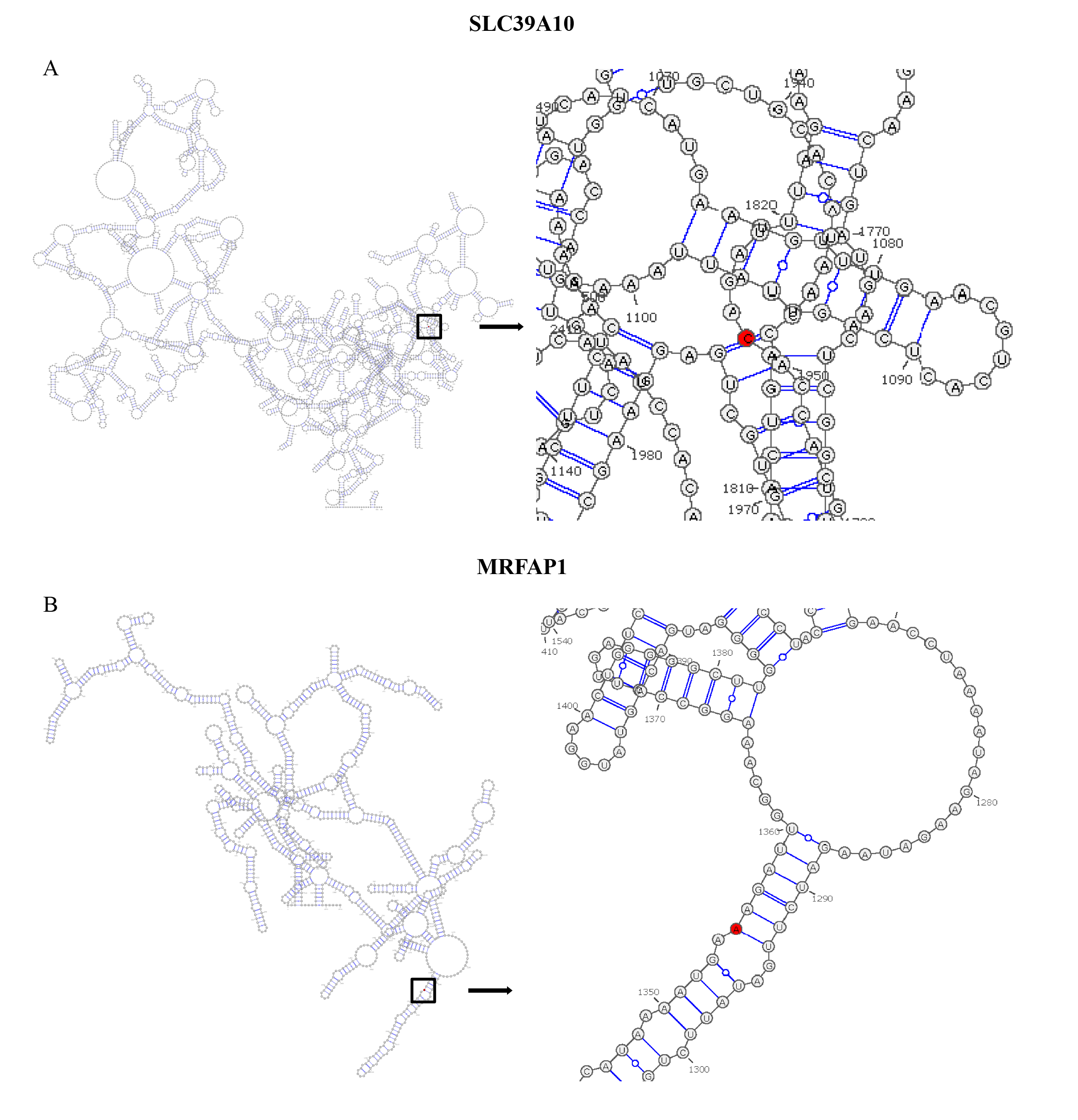
**

**Figure S4. 2'-O-methylation predicted site.** 2'-O-methylation predicted site plots of SLC39A10**(A)** and MRFAP1**(B)** obtained via the DeepOMe website.


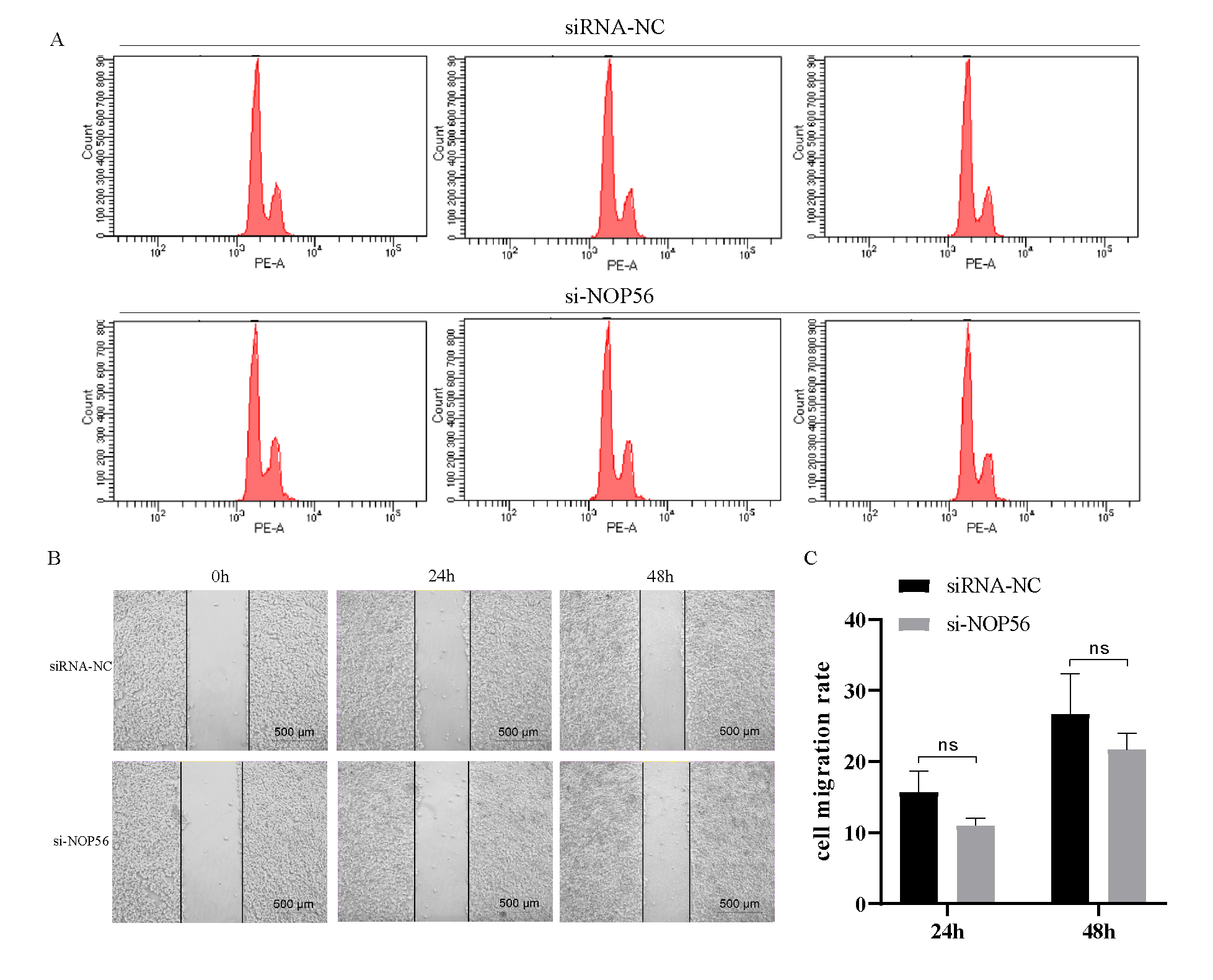


**Figure S5. Effect of knockdown of NOP56 on HepG2 cell cycle and migratory capacity. (A)** Cell cycle situation of HepG2 cells after knockdown of NOP56. **(B, C)** Migratory capacity of HepG2 cells after knockdown of NOP56.
